# Cottage Hospitals and General Practitioners

**Published:** 1895-02-16

**Authors:** 


### COTTAGE HOSPITALS AND GENERAL PRACTITIONERS.

Ever since the late Mr. Napper founded the first cottage hospital at Cranley, in Surrey, in the year 1859, patients admitted to cottage hospitals have been entitled to receive the medical attendance of their own medical men, whenever he has been a duly qualified practitioner resident in the district. Here and there cases have occurred where an individual member of the profession has, so to speak, attempted with more or less success to capture a cottage hospital, and sometimes in defiance of the rules to set himself to deprive the patients of this privilege. A recent interesting instance has occurred in Wales, where the rules of the cottage hospital provide that any medical practitioner resident within a defined area, possessing the qualifications required by the Local Government Board, shall be allowed to visit his own patients in the hospital. The intention of the rule, which is a common one, as we have already stated, in cottage hospitals, is that each patient shall receive at his option the medical attendance of his own medical man. In spite of the rule an attempt has been successfully made by one of the medical officers of the cottage hospital in question to deprive the patients of the services of their own medical attendant. Whilst desirous of keeping ourselves free from all local disputes on hospital matters, we cannot help stating in the public interest that the proceedings of the committee in question, as they have been reported to us, are contrary to all precedent and practice, so far as we know anything of cottage hospital management, and cannot be upheld on the principles of sound administration or justice. The interests of the patients, and consequently of every hospital, demand that the rule just quoted shall be interpreted to mean, what it clearly was intended to mean—namely, that each medical practitioner of the district shall have the privilege, if he wish it, of treating his own patients in the cottage hospital. This system has worked admirably in cottage hospitals all over the country, and we hope the chairman of the committee will be supported by the governors and their colleagues in upholding the rule and its clear interpretation. Of course it is open to any governor to move that this particular rule be rescinded, but such a step would be contrary to the best interests of the patients and the institution, apart from all local feeling or controversy, and we therefore urge the governors to maintain this rule and see that it is henceforth interpreted in a liberal spirit.

